# Structural Behavior of Fibrous-Ferrocement Panel Subjected to Flexural and Impact Loads

**DOI:** 10.3390/ma13245648

**Published:** 2020-12-11

**Authors:** Gunasekaran Murali, Mugahed Amran, Roman Fediuk, Nikolai Vatin, Sudharshan N. Raman, Gundu Maithreyi, Arunachalam Sumathi

**Affiliations:** 1 School of Civil Engineering, SASTRA Deemed to be University, Thanjavur 613404, India; maithreyi97@gmail.com (G.M.); sumathi@civil.sastra.edu (A.S.); 2 Department of Civil Engineering, College of Engineering, Prince Sattam Bin Abdulaziz University, Alkharj 11942, Saudi Arabia; 3 Department of Civil Engineering, Faculty of Engineering and IT, Amran University, Amran 9677, Yemen; 4 School of Engineering, Far Eastern Federal University, 8, Sukhanova Str., Vladivostok 690950, Russia; roman44@yandex.ru; 5 Higher School of Industrial, Civil and Road Construction, Peter the Great St. Petersburg Polytechnic University, 195251 St. Petersburg, Russia; vatin@mail.ru; 6 Civil Engineering Discipline, School of Engineering, Monash University Malaysia, Selangor 47500, Malaysia; sudharshan.raman@monash.edu

**Keywords:** ferrocement, flexure, impact, fiber, mesh, crack width, failure mode

## Abstract

Ferrocement panels, while offering various benefits, do not cover instances of low and moderated velocity impact. To address this problem and to enhance the impact strength against low-velocity impact, a fibrous ferrocement panel is proposed and investigated. This study aims to assess the flexural and low-velocity impact response of simply supported ferrocement panels reinforced with expanded wire mesh (EWM) and steel fibers. The experimental program covered 12 different ferrocement panel prototypes and was tested against a three-point flexural load and falling mass impact test. The ferrocement panel system comprises mortar reinforced with 1% and 2% dosage of steel fibers and an EWM arranged in 1, 2, and 3 layers. For mortar preparation, a water-cement (w/c) ratio of 0.4 was maintained and all panels were cured in water for 28 days. The primary endpoints of the investigation are first crack and ultimate load capacity, deflection corresponding to first crack and ultimate load, ductility index, flexural strength, crack width at ultimate load, a number of impacts needed to induce crack commencement and failure, ductility ratio, and failure mode. The finding revealed that the three-layers of EWM inclusion and steel fibers resulted in an additional impact resistance improvement at cracking and failure stages of ferrocement panels. With superior ultimate load capacity, flexural strength, crack resistance, impact resistance, and ductile response, as witnessed in the experiment program, ferrocement panel can be a positive choice for many construction applications subjected to repeated low-velocity impacts.

## 1. Introduction

Many countries in the developing world, technological advancement towards mass housing development directed towards use of imported material to a great extent. With the huge demand for housing, the available source of traditional material for construction like cement, fine aggregate, coarse aggregate, and steel are likely to deplete at accelerating rates, unless these are used judiciously. To provide necessary housing infrastructure to millions of people without shelter, developing cost-effective material for buildings has become an immediate necessity. Available research recommends that ferrocement panels be an effective substitute, as it is cost-efficient [1]. Ferrocement is a unique sort of thin reinforced composite developed of cement mortar [2] in which one or several wire mesh layers are used as the primary reinforcement. In 1848, Joseph Louis Lambot was invented and conceived originally for boat building [3]. Even as ferrocement earned widespread use in the mid-20th century, including in the building sector, additional benefits have been identified over traditional reinforced concrete. A series of benefits are: (i) the simplicity of development owing to less necessary expertise in creation; (ii) economic feasibility owing to a lower expenditure of base materials and human resources; (iii) superior flexural behavior due to increased surface of reinforcement; (iv) capacity to be fabricated in an assortment of shapes, like barrels, shells, arches, and alike; (v) appropriateness of material for repair and retrofitting of structures that already exist; (vi) higher tensile strength to weight ratio because of less unit weight; and (vii) higher cracking performance, suited to water retaining structures in particular.

As a result of many benefits over conventional reinforced concrete, ferrocement uncovers broad implementation in partitioning structures, waste bins, footbridges, roof shells, silos, swimming pools, manhole covers, inexpensive modular housing, and so forth. The utilization of ferrocement is standard across the globe, but it is increasingly valued in industrialized nations because of its economic profitability and requires less skilled laborers. Numerous applications of ferrocement include typical structural elements of building [4], permanent formwork for concrete members [5], floors [6], roofing elements [7], water tanks [8], structural retrofitting [9], and boats [10].

There has been plenty of research on the flexural performance of ferrocement employed with waste material as cement binder and sand utilized to form matrix. Studies focused on the structural performance of fibrous ferrocement and efficiency in fibers for developing structural ferrocement are scarce. Much research has focused on using waste from the industry as a partial substitution of cement. For instance, Memon et al. [11] studied cement in ferrocement being replaced by 50% and 60% of ground granulated blast furnace slag. Findings indicated that the high amount of slag in ferrocement exhibited sufficient flexural strength and workability for the thin ferrocement construction to resist structural loading. Al-Kubaisy and Jumaat [12] examined the flexural behavior of ferrocement with varying thickness and wire mesh volume fraction. The findings revealed that utilizing ferrocement is greatly diminishes deflection, the crack’s width, a spacing of crack at ultimate loads. Sakthivel and Jagannathan [13] investigated the flexural behavior of ferrocement panels made with galvanized iron mesh coated by polyvinyl chloride. The size of the panel used in this study was 700 mm × 200 mm × 15 mm. The wire mesh configuration and the number of reinforcing mesh layers are the variables investigated. The research suggested that the wire mesh panel coated with polyvinyl chloride displayed less strength by about 10% than panels with uncoated galvanized iron mesh. Kulkarni et al. [14] investigated the influence of the various thicknesses of ferrocement panels on flexural behavior. Findings indicated that crack initiation and ultimate crack rely on the number of reinforcing mesh and thickness in the ferrocement panels. The flexural response of ferrocement panels incorporated with fly ash was studied against the acidic environment Chandrudu and Desai [15]. The panel size considered in the study was 970 mm × 300 mm × 35 mm, and all panels were tested against four-point bending. The parameters considered were quality of mortar, curing environment, and period of exposure and number of reinforcing mesh layers. It results that the optimum dosage of fly ash was observed by 10%. Furthermore, it was noted that a rise in hydrochloric acid concentration diminished the flexural strength of ferrocement panels.

Besides, the outcome of some experiments aimed at producing ferrocement with different mesh together with fibers. The impact of cement substitution by different silica fume contents in ferrocement panels of size 500 mm × 200 mm × 50 mm was explored by Mousavi [16]. Galvanized wire mesh was used to reinforce first, second, and third layers together with steel fibers to prepare ferrocement panels. A three-point flexural laboratory test was performed on all simply supported panels. The studies’ findings indicated that the flexural strength increased by about 3.6 times in ferrocement panel comprising 15% silica fume and 4% fibers compared to conventional mortar. Superior resistance to crack and enhanced flexural capacity was observed from formulated optimum mix design comprised 85% of plain cement, 15% of silica fume, 4% of steel fiber with three-layer wire mesh. Mughal et al. [17] studied the potential use of polypropylene and galvanized iron meshes as reinforcement in ferrocement panels. Results revealed that the ratio of the ultimate load of galvanized iron mesh panel to the polypropylene mesh panel was up to 3.8 times for the flexural strength test. Higher initial stiffness in the galvanized iron mesh panel was observed than the polypropylene mesh panel. The ultimate load in the flexural strength tended to rise with the rise in the panel’s thickness and the number of mesh layers. Shaheen et al. [18] studied the possible application of ferrocement concrete to restore the deteriorated panels that collapsed against impact load. Ferrocement panels of dimension 500 mm × 500 mm × 20 mm were prepared with expanded steel mesh and welded galvanized mesh and tested up to failure. The deteriorated panels were renovated using galvanized steel mesh provided at the panel’s top and bottom faces with the help of a shear connector. Results indicated that rehabilitated panels result in improved energy engrossment, ductility ratio, and better cracking configuration attained without falling of concrete cover. The paper presents results from the current study that assesses the flexural and impact response of ferrocement panels made with steel fibers and expanded wire mesh.

## 2. Significance of Research

The ferrocement panel system’s flexural behavior made with different admixtures and wire meshes has already been investigated; however, findings on low-velocity impact behavior of ferrocement panel incorporated fibers are scarce. The current research aims to fill gaps in the research thus far. In this pilot research, the above-mentioned inadequacies have been addressed by producing a fibrous ferrocement panel with expanded wire mesh and assessing their flexural and impact performance. Hooked steel fibers were added at 1% and 2% dosage with 1, 2, and 3 layers of expanded wire mesh to assess ferrocement panels’ potential. The study is expected to provide valuable insights for the formulation and implementation of fibrous ferrocement panels subjected to low-velocity impact.

## 3. Experimentation Program

### 3.1. Raw Materials

**Figure 1:**
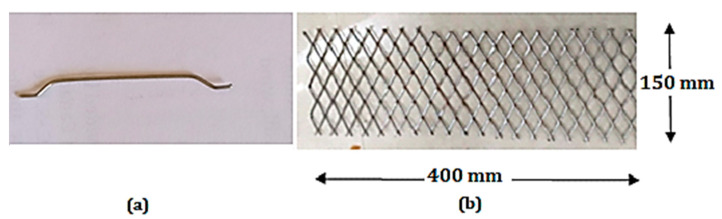
Details of fiber and EWM (**a**) Geometric shape of steel fiber and (**b**) EWM utilized in ferrocement panel under study.

The necessary raw materials utilized in this study were purchased from domestic sources. Ordinary Portland cement was utilized as a binder as per American society for testing and materials (ASTM C 642–82) types I [19] and IS 12,269 [20] for producing the ferrocement matrix. The required well-graded river sand was procured locally, having a fineness modulus of 2.45, a nominal maximum size of 4.75 mm, and a density of 2600 kg/m^3^ for use in the mortar mixes. The water to binder (w/b) ratio was maintained as 0.4, and the ratio of binder to sand was 1:2 by weight. High range super plasticizing admixture (Polycarboxylic ether) was utilized to decrease water in the mortar mixes. The dosage of super plasticizing admixture varied from 0.7 to 1.3 by weight of cement. The relative density super plasticizing admixture from the supplier is 1.08 ± 0.01 at 25 °C and the pH values were 7−9. For mixing and curing of ferrocement panel, ordinary locally available portable water was utilized. Expanded wire mesh (EWM), easily obtainable in the local market, was used as reinforcement in the ferrocement panel. According to manufacturer data, the mechanical properties of expanded wire mesh as follows: 15 mm × 30 mm of dimensions size, 1660 gm/m^2^ of weight, 1.5 mm thickness of the sheet, 12 × 10^3^ MPa of Young’s modulus, 250 MPa of yield stress, 9.7 × 10^3^ of yield strain, 380 MPa of ultimate strength, and 59.2 × 10^3^ of ultimate strain. Steel fiber with hooked ends was utilized in this study, where the diameter of the fiber is equal to 0.6 mm, the length of fiber is 30 mm, and the tensile strength is 1100 MPa. The geometric shape of steel fiber with hooked-end and expanded wire mesh was utilized in the ferrocement panel, as shown in Figure 1.

### 3.2. Mortar Matrix and Mix Composition

The cement mortar utilized for producing panels was designed to attain a compressive strength of 25 MPa at 28-days. The properties of mortar mixes were selected in conformity with the American concrete institute (ACI committee 549) reports [21]. The amount of water and water-reducing agents were altered in the mixes to achieve 180–200 mm mortar flow in line with the recommendation of ASTM C 1437–99 [22]. The laboratory mechanical mixer was utilized to formulate mortar for all the mixes. First, the cement and sand were dry-mixed for 2 min; second, portable water mixed thoroughly with a water reduction agent, and then the entire batch was remixed for 2 min. Finally, steel fibers with hooked ends were dispersed in the mortar mix, and mixing was continued until uniform consistency was achieved. The mixing composition of cement, sand, water, water-reducing admixtures and fiber dosage is given in Table 1.

### 3.3. Preparation of Specimen

**Figure 2:**
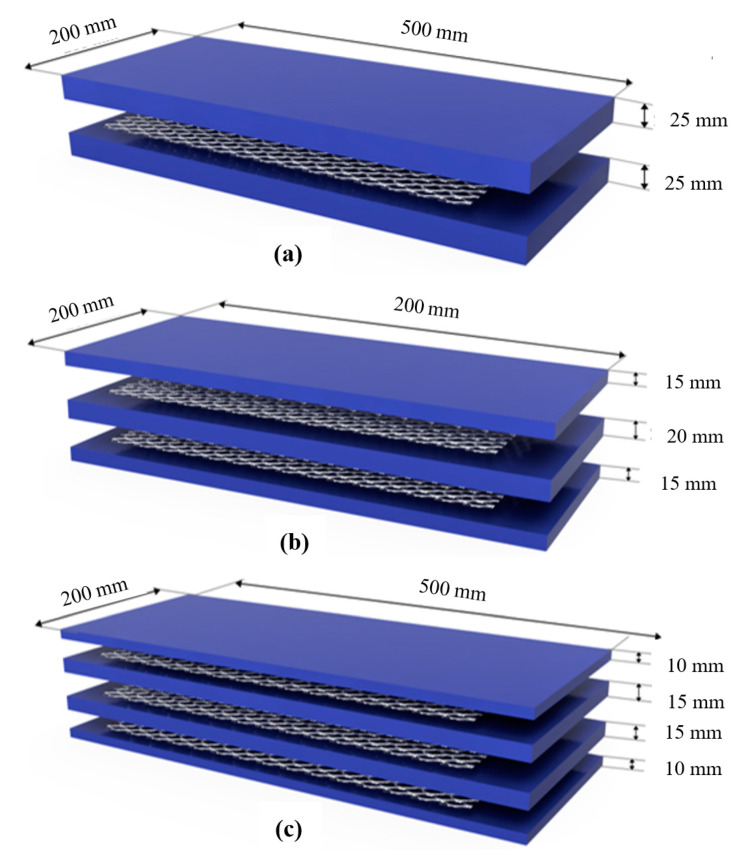
Details of layer thickness (**a**) single mesh; (**b**) double mesh; and (**c**) triple mesh.

To assess the flexural and impact strength of ferrocement panels, twelve mixes were used with the combined action of steel fiber and expanded wire mesh. Two different fiber dosages and three different expanded wire mesh layer schemes were used in this study. The first mix was designated as FP-0-0, where FP denotes the ferrocement panel, the first 0 denotes a layer of expanded wire mesh, and the second zero denotes the dosage of steel fiber. The second mix was designated as FP-0-1, where FP denotes the ferrocement panel, 0 denotes a layer of expanded wire mesh, and 1 denotes that a 1% dosage of steel fiber has been used. Likewise, the other specimens were also designated in the same sequence, as discussed earlier. It is worth pointing out that the mixes 1−3, 4−6, 7−9, and 10−12 comprised 0, 1, 2, and 3 layers of EWM, respectively. The details of the panel layer thickness for a different scheme of reinforcement are given in Figure 2.

**Figure 3:**
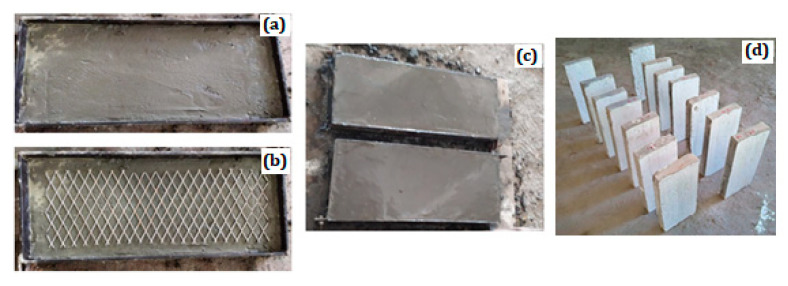
Process of casting ferrocement panel (**a**) finished layer before mesh placement; (**b**) mesh arrangement; (**c**) finished ferrocement panel; and (**d**) ferrocement panel before testing.

To construct the first layer of ferrocement panel for the mixes (FP-1-0, FP-1-1, and FP-1-2), mortar was applied at designed layer thickness and allowed to attain some initial setting. Subsequently, the expanded wire mesh is placed on top of first layer, accompanied by the second layer of mortar, as shown in Figure 3. For the mixes (FP-2-0, FP-2-1, and FP-2-2), the procedure mentioned above was adopted to complete three layers, including the expanded wire mesh placement above the first and second layers. For the mixes (FP-3-0, FP-3-1, and FP-3-2) comprised of three-layer reinforced schemes, four layers were constructed one by one with expanded wire mesh placement above the first, second, and third layers. All panels were cast in steel formwork, and care was taken to finish the panel’s smoothed top surface. Twelve different ferrocement panels of size 500 mm × 200 mm × 50 mm were prepared and subjected to immersion curing for 28 days before the scheduled tests. 

### 3.4. Test Setup

**Figure 4:**
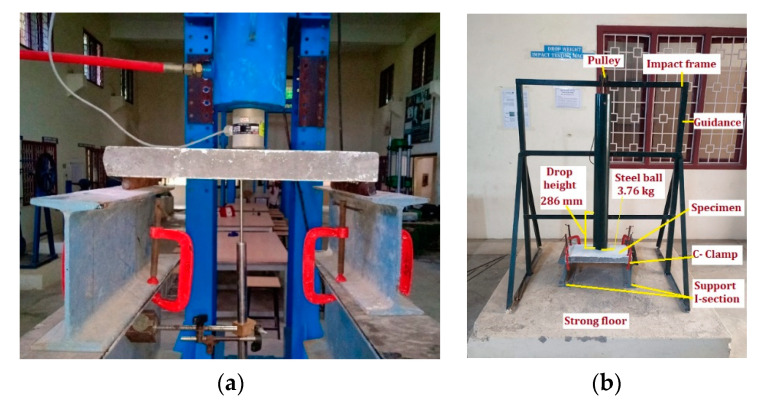
Test setup (**a**) flexure and (**b**) impact.

Three specimens were prepared for each mix and tested; the mean values of test results were reported. The compressive strength of 100 mm cubical non-fibrous and fibrous specimens was tested conforming to IS 516 [23]. All specimens were tested using 300 T capacity compression testing machine (India). A three-point flexure test was done using a displacement control loading frame in accordance with ASTM C1609–12 [24]. Self-balancing loading frame with 25 T compression load cell and 30 T hydraulic remote jack was used to test all ferrocement panels. Ferrocement panels were accurately fixed with the use of C-clamp to prevent the movement of the support while loading (I section). Figure 4a illustrates the testing setup for flexural strength. The ferrocement panels spanned 400 mm and tested at a 2 mm/min loading rate. The mid-span deflection was recorded with linear variable displacement transducers (LVDT) (Tamilnadu, India), which was placed at mid-bottom face. The load and corresponding deflection up to the ultimate load were recorded automatically by 16 channel data acquisition system. The portable digital microscope was used to measure the width of the crack corresponding to the ultimate load. The flexural strength of the ferrocement panel was calculated by Equation (1).

(1) $$\sigma =\frac{WL}{bd^{2}}$$

where,

*σ* = flexural strength, MPa
*L* = support span, mm
*b* = tested panel width, mm
*d* = tested panel depth, mm
*W* = applied load, N

A falling mass impact test was conducted on panels using the falling weight apparatus shown in Figure 4b. The falling mass test was done based on the ACI Committee 544 [25] recommendations. The test set up comprises a steel ball that weighs 3.76 kg, lifted to a vertical distance of 286 mm from the target specimen’s top surface. The test is conducted by the gravity dropping of the ball on the target repeatedly. The target specimen is kept in place with no motion during the test using C clamps. All panels with a 400 mm span are struck with falling weight on its top face’s center. The impact response is determined visually using the number of impacts leading to cracking (Z1) and failure (Z2) of the target specimens. Equation (2) defines the used simplified method to calculate the absorbed energies at cracking (P1), and failure (P2) cases using the recorded numbers of impacts.

Impact energy (P1 or P2) = N × m × g × H(2)

where, N: recorded impacts number, m: 3.76 kg (steel-ball weight), g: gravitational acceleration (9.81 m/s^2^), and H: vertical falling distance.

## 4. Discussion of Results

### 4.1. Compressive Strength

**Figure 5:**
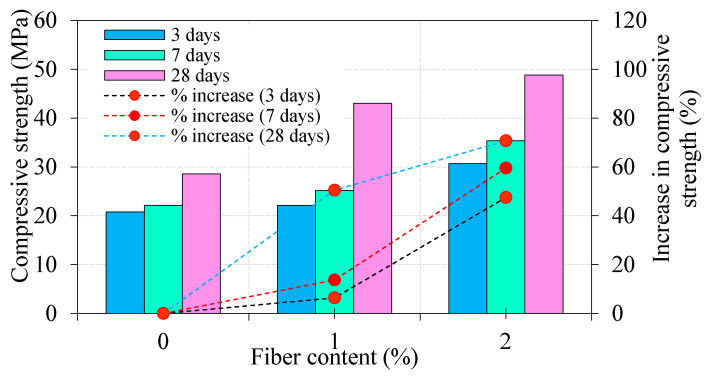
Effect of steel fiber content on the compressive strength of mortar.

Fiber characteristics affect the development of the compressive strength of cement mortar over time. It can be seen that the compressive strength of steel fiber used to cement mortar was enhanced in the early days at a higher dosage of fiber. The effect of the steel fiber dosage on the compressive strength of mortar cube specimens is shown in Figure 5. It shows that the addition of fibers increased the compressive strength than the non-fibrous specimen. The addition of 1% and 2% dosage of fiber leads to an increase in compressive strength by about 6.4% and 47.5% at 3 days, respectively, compared to non-fibrous specimens. Further increase in compressive strength was observed by about 13.7% and 59.5% at 7 days and 50.5% and 70.8% at 28 days. With fibers, the instigation of cracks due to tensile stress and possible shear stress appear to be delayed and also appear postponed. This phenomenon is because when a crack meets a fiber, it requires more energy of fracture to drag the fiber out and then extend. Therefore, the relative limit and ultimate strength of the mortar cubes are generally augmented; this is intensely reliant on the fibers’ matrix, stiffness, and dispersion. So that if the interfacial bond is adequate, the surrounded matrix between the fibers will be restricted, and subsequently, a higher strength capacity is attained. Findings indicated that the increase in compressive strength is influenced by the content of fiber in the specimens. In the nutshell, the addition of steel fibers also contributed to the strength enhancement of the test specimens. This is due to the bonding interaction between steel fibers and mortar paste. This bonding at fiber–mortar matrix plays a significant role in compressive strength performance of cubical specimens. According to Alvarez et al. [19], after occurring fracture of the test specimens, additional load could be supported by the bonding of the fibers and mortar paste before the separation specimen.

### 4.2. Load-Deflection Behavior

**Figure 6:**
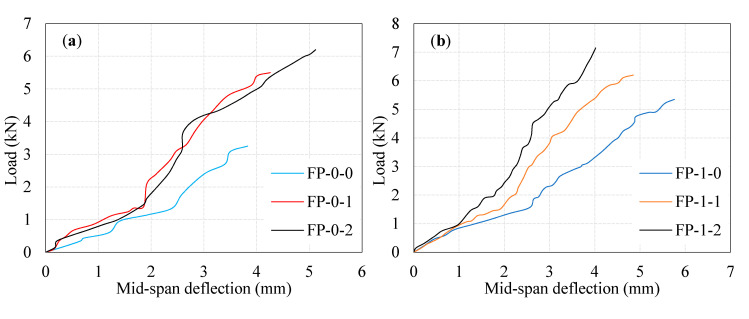

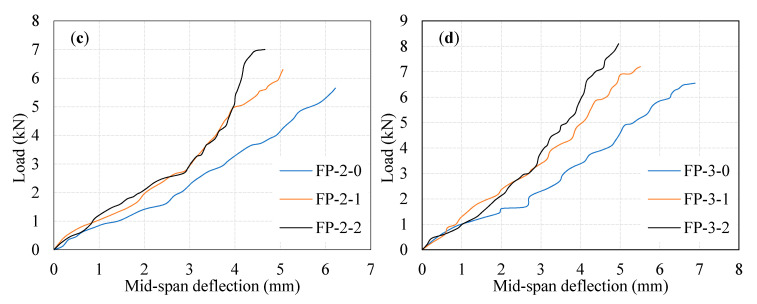
Load-mid span deflection profiles. (**a**) zero layer mesh; (**b**) one layer mesh; (**c**) two layer mesh and (**d**) three layer mesh.

The deflection of the ferrocement panel under three-point loading is essential in its. Figure 6a–d illustrate the curves of load versus mid-span deflection of tested ferrocement panels. Two different deflection phases were noticed in all tested panels: before cracking in a mortar, and reinforcement yielding. The first phase of deflection corresponding to the panel remains un-cracked. The second phase describes the post-yielding phase [26]. The curves of load versus mid-span deflection of the tested ferrocement panels in this study resemble those stated in past investigations [27,28,29,30,31]. It can be commonly noticed that the slope of load-mid-span deflection curves is steep initially, but decreases gradually, then turns into zero at the ultimate loading point. The same pattern is reported in another investigation [32].

Additionally, it is noticed that increasing the number of EWM layers in the ferrocement panel is accompanied by the increased load carrying capacity, reduced mid-span deflection with increased ductility. For the FP-0-0, FP-0-1, and FP-0-2 panels, the observed first crack load was 1.15, 1.40, and 1.45 kN with the corresponding deflection was 3.25 mm, 5.50 mm, and 6.20 mm respectively. It can be noticed prudently from Figure 6a that the first crack load increased with fiber dosage. This phenomenon may be attributed to the presence of fiber, which leads to bridging action during tensile stress transfer resulting in a higher load at first crack. In one-layer EWM panels (FP-1-0 to FP-1-2) had a positive contribution in the first crack load ranged from 1.56–1.62 with the corresponding deflection values in the range of 5.35–7.15 mm. A minimal effect on the first crack load was observed in two-layer EWM panels (FP-2-0 to FP-2-2) compared to one-layer EWM panels. The observed first crack load varied from 1.6 to 1.78 kN, with the corresponding deflection values ranging from 5.65–7.0 mm. The maximum first crack strength was observed in three-layer EWM panels (FP-3-0 to FP-3-2) in the range of 1.71–1.83 kN with the corresponding deflection varying between 6.55 mm and 8.1 mm. It is observed that the number of layers of EWM and steel fibers had a small contribution to the increase in first crack load in the range of 36% to 59% as compared to FP-0-0 panel.

In the post yielding of the reinforcement phase, the increment in the ultimate load and the corresponding mid-span deflection rate for all tested ferrocement panels are shown in Table 2. From Figure 6a–d, it may be noted that the significant increase in the ultimate load of the ferrocement panel due to increasing the number of EWM layers and fiber dosage. For FP-0-1 and FP-0-2 panel, the observed ultimate load was 5.5 and 6.2 kN with the corresponding deflection 4.26 mm and 5.12 mm. Findings indicated that 1% and 2% fiber addition leads to a high increase in the ultimate load capacity of about 69% and 91%, respectively. The reason for this behavior is the same as discussed earlier in Figure 6a. In single-layer EWM panels, the FP-1-0 panel exhibited a 65% higher ultimate load than its FP-0-0. Simultaneously, the ultimate load of FP-1-1 and FP-1-2 panels was increased by about 91% and 120%, respectively. The mid-span deflection corresponding to these panels’ ultimate load was observed in the range of 5.76–4.02 mm, as shown in Figure 6b. This phenomenon is due to the matrix fracturing, fiber bridging, and one layer of EWM yielding simultaneously resulting in higher ultimate load carrying capacity and lower deflections. However, the maximum ultimate load connected with the corresponding mid-span deflection of the FP-1-2 panel shows an impressive increase is a stiffness.

The utilization of double-layer EWM, together with fibers, significantly increases the ultimate load capacity and reduces the deflection. The inclusion of EWM from one to two layers increases the ultimate load by 6%, 2%, and 3% for the FP-2-0, FP-2-1, and FP-2-2 panels compared with FP-1-0, FP-1-1, and FP-1-2 panels, respectively. The same panels’ ultimate load was increased by about 74%, 94%, and 115% compared to the reference panel (FP-0-0). The corresponding deflection of these panels was in the range of 6.22–4.66 mm, significantly reduced with increasing fiber dosage. The deflection depends on the number of EWM layers, fiber dosage, and properties of the matrix. It may be concluded that the ferrocement panel, with the inclusion of two layers of EWM + fibers exhibited a higher load carrying capacity compared to those with one-layer EWM together with fibers. This phenomenon is attributed to the following three reasons: (i) Strength of cement matrix. (ii) Fiber with hooked end tends to produce higher bonding with the matrix surrounding the fibers, leading to greater pull-out strength. This behavior dramatically improves the behavior of crack prevention. The effective tensile stress transfer occurs across the cracked regions, resulting in higher load capacity and (iii) two layers of EWM placed at two different thickness locations, which leads to acting as a barrier during the crack proliferation delaying failure.

In the three-layers EWM in ferrocement panels, the ultimate load capacity increased significantly compared to one-layer and two-layers EWM panels. The FP-3-0, FP-3-1, and FP-3-2 panels’ ultimate load capacity were increased by about 102%, 122%, and 149%, respectively, compared to FP-0-0 panel. The observed deflection corresponding to the ultimate load was in the range of 6.89–4.96 mm. The ultimate load capacity increased in the range of 13–22% compared to single-layer EWM and fiber panels, while in the range of 10–16% increment was observed compared to two-layers EWM and fiber panels. This behavior implies that EWM and fibers’ combined action positively contributed to an increase in ultimate load capacity and reduced deflection. It is worth pointing out that the best contribution comes in terms of EWM inclusions was three-layers EWM panels followed by two-layer and one-layer. The reason behind this phenomenon has already been indicated in earlier discussions. Furthermore, another reason for this behavior is the placement of EWM at a certain distance. This behavior is in proper alignment with the earlier study of Baston et al. [33].

The ductility index is defined as the deflection ratio at the ultimate load to deflection at crack initiation. As shown in Table 2, the ductility index values range from 2 to 2.8 for FP-0-0 to FP-0-2 panels, 2.3 to 2.8 for FP-1-0 to FP-1-2 panels, 2.5 to 2.9 for FP-2-0 to FP-2-2 panels, and 2.6 to 2.9 for FP-3-0 to FP-3-2 panels. This indicates the increasing number of EWM layers with increasing fiber dosage, leading to a higher ductility index. Arguably, the number of warnings a ferrocement panel is occurred before failure due to superior in three-layer EWM panel with a 2% fiber dosage.

### 4.3. Combined Effect of Fiber and EWM on Flexural Strength

**Figure 7:**
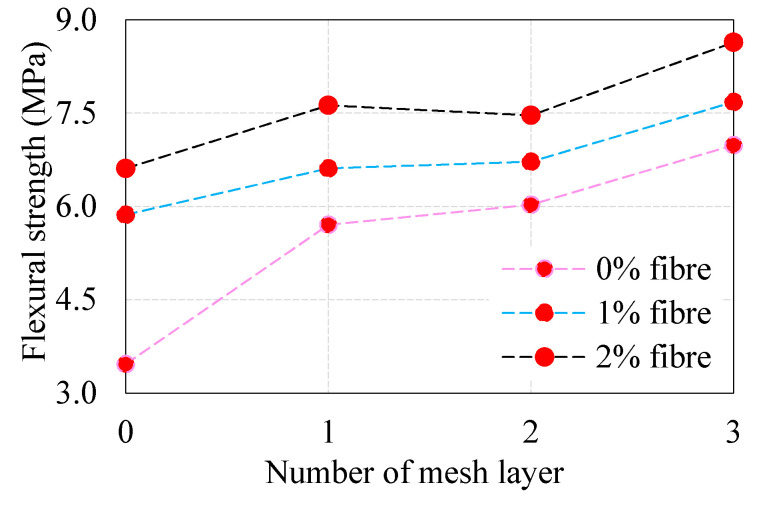
Combined effect of fiber and EWM on flexural strength of ferrocement panel.

Figure 7 shows the combined effect of fiber and different numbers of EWM layers on the flexural strength of 28 days age specimens. Adding 1% and 2% dosage of fibers shows the most excellent effect of ferrocement panel (FP-0-1 and FP-0-2), by 69% and 91%, respectively, compared to FP-0-0 panel. As seen in Figure 7, the increasing number of EWM increases flexural strength. For example, the increase in flexural strength of FP-1-0, FP-2-0 FP-3-0 panel with respect to FP-0-0, is about 65%, 75%, 104%, respectively. It is worth observing from Figure 7, the FP-1-1, FP-2-1 FP-3-1 panels showed about 13%, 15%, and 31% increase in flexural strength, respectively, compared to FP-0-1 panel, respectively. This effect may be explained by the concentration of stresses between the two layers of EWM, while an excess of fiber in this space only enhances this positive concentration. However, the situation changes with the introduction of the third layer of EWM. In this case, there is a decrease in crack formation rate and a decrease in the concentration of stresses around the fiber; accordingly, the stresses in the ferrocement panel structure are balanced and redistributed between the structural components of the panel. Regardless of the amount of fiber added, or even if there is no fiber, the flexural strength graph has the same linear behavior with introducing the third EWM layer. The observed flexural strength of FP-1-2, FP-2-2, FP-3-2 panels were increased by about 15%, 19%, and 31%, respectively, compared to the FP-0-2 panel. It is clear from the above discussions, the flexural strength responded positively to increasing fiber dosage and the number of EWM layers. This phenomenon is attributed to the mortar matrix’s better compaction, uniform distribution of fiber, and EWM placed an appropriate distance apart.

### 4.4. Comparison of Crack Width

**Figure 8:**
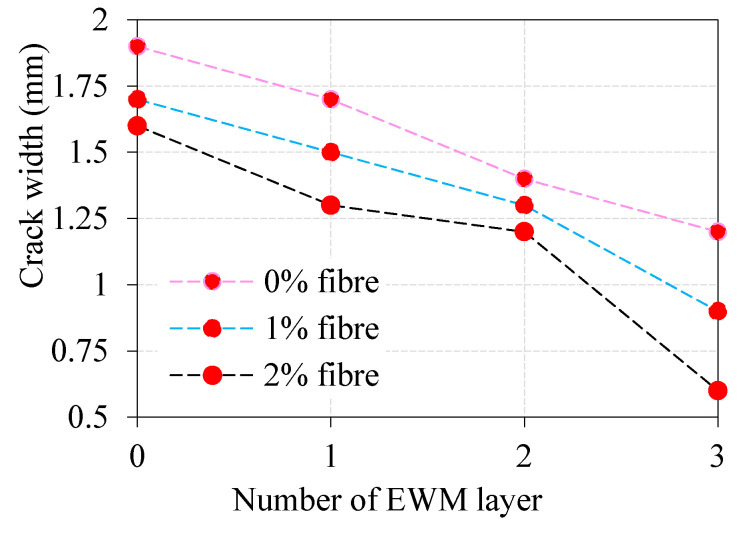
Comparison of crack width of ferrocement panels with various fiber dosage and EWM layers.

A study of the combined effect of different dosages of steel fiber and a different number of EWM layers on the crack opening width of 28 days age specimens is shown in Figure 8. The curves of the crack opening dynamics resemble the curves of changes in flexural strength shown in Figure 7. However, there are specific differences between these curves. The observed crack width of FP-0-0, FP-0-1 and FP-0-2 were 1.9 mm, 1.7 mm and 1.6 mm, respectively. Adding one layer of EWM in ferrocement panel (FP-1-0, FP-1-1, and FP-1-2) reduces the crack opening width by 11%, 12%, and 19%, respectively, with respect to FP-0-0, FP-0-1, and FP-0-2 panels. Thus, the maximum positive effect is achieved by increasing the amount of fiber introduced at the stage of structure formation, by redistribution of stresses during plastic shrinkage from the most dangerous zones to the entire volume of the ferrocement panel. This is due to the fact that at the stage of structure formation, stress redistribution occurs during plastic shrinkage from the most dangerous zones to the entire volume of the ferrocement panel. Simultaneously, when adding a second layer of EWM, reducing the crack openings width becomes the opposite decreasing with an increased fiber dosage. For FP-2-0, FP-2-1, and FP-2-2 panels, the crack opening width decreases by 26%, 24%, and 25%, respectively, with respect to FP-0-0, FP-0-1, and FP-0-2 panels. Additionally, as it was already revealed in previous tests, the use of a three-layer EWM gives the optimal crack containment effect. Compared to ferrocement panels with two-layer EWM, the addition of a third layer of EWM improves the composite’s crack resistance by 37%, 53%, and 68%, respectively, for FP-2-0, FP-2-1, and FP-2-2 panels with reference to reference panels discussed above. The width of crack opening up to 0.6 mm observed, in this case, allows us to speak of the developed ferrocement panels with three-layer EWM and 2% steel fiber, as rather crack-resistant composites.

### 4.5. Impact Strength Results

The recorded number of impacts leading to cracking (Z1) and failure (Z2) of the target specimens and its corresponding impact energy at cracking and failure stage are summarized in Table 3.

#### 4.5.1. Effect of Fibers on Impact Strength

**Figure 9:**
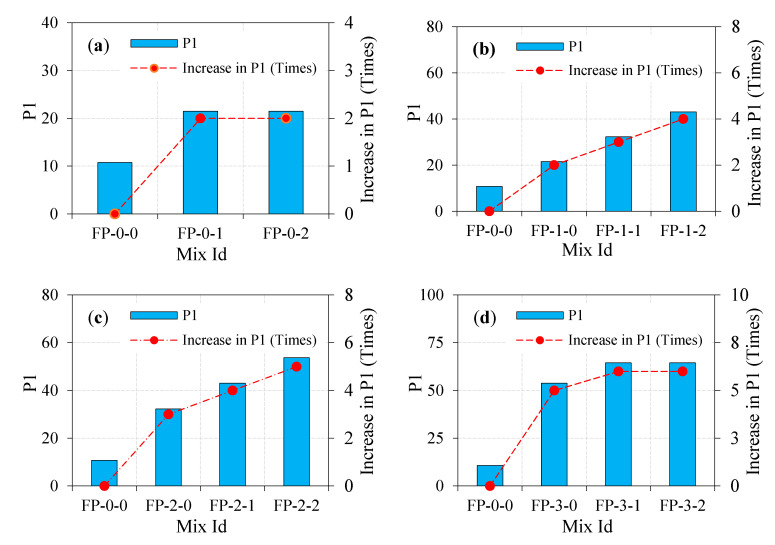
Number of impacts needed to induce crack commencement. (**a**) zero layer mesh; (**b**) one layer mesh; (**c**) two layer mesh and (**d**) three layer mesh

**Figure 10:**
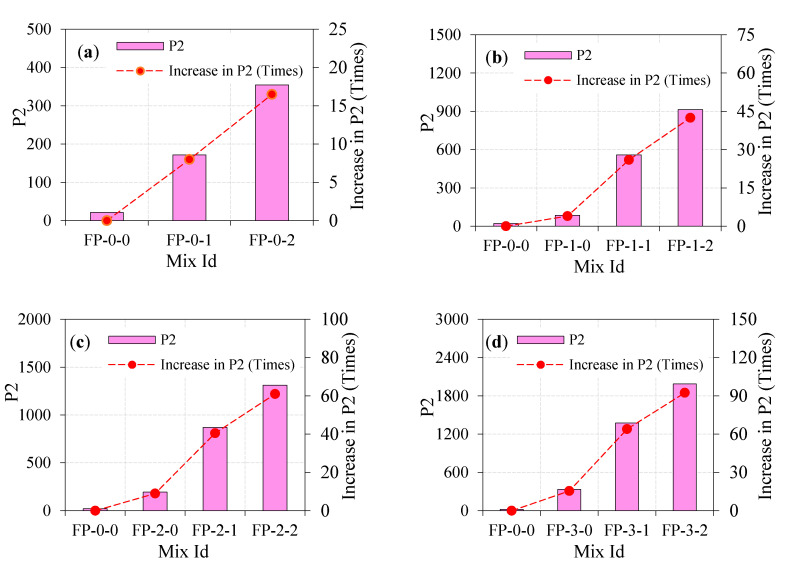
Number of impacts needed to induce failure. (**a**) zero layer mesh; (**b**) one layer mesh; (**c**) two layer mesh and (**d**) three layer mesh

The impact resistance of ferrocement panel in terms of the number of impact leading to cracking with the dosage of steel fibers and the number of impact leading to failure are shown in Table 3. The impact resistance of the ferrocement panel improves with the increase of fiber dosage. For the FP-0-0 panel, the engrossed impact energies at the cracking stage (P1) and the failure stage (P2) were 10.7 J and 21.5 J, respectively. By comparing FP-0-1 with FP-0-0 panel, it is noted that adding a 1% dosage of fiber increased P1 and P2 by 2 and 8 times, respectively. Simultaneously, the inclusion of a 2% dosage of steel fiber increased P1 and P2 by 2 and 16.5 times, respectively. This phenomenon highlights the high-potential impact energy absorption due to the inclusion of fiber. This is imputed to the fiber bridging action for the period of transferring load across the crack leading to hinder or downturn the crack growth, thereby increasing the required number of impacts [34]. This is an anticipated affirmative measure of fibers that can enhance the tensile strength through crack control, leading to more ductile behavior against impact loads [35]. It also indicates that, in general, a panel comprising a higher dosage of steel fiber exhibited higher energy engrossment compared to the panel without fiber (see Figure 9a and Figure 10a), demonstrating the favorable use of steel fibers in ferrocement panels.

#### 4.5.2. Combined Effect of Fibers and a Single Layer of EWM on Impact Strength

Figure 9b and Figure 10b illustrate the engrossed impact energies at the cracking and failure stage for the panel comprising fibers and one layer of EWM. It is quite evident from the figures that the addition of fiber together with EWM increases impact energies dramatically by greater than or equal to 2 times in the worst-case scenario compared to FP-0-0 panel, while this higher energy reaches to 42.5 times. Considering Figure 9b and Figure 10b, the impact energies P1 and P2 for the FP-1-0 panel were 2 and 4 times higher, respectively, compared to the FP-0-0 panel. These values display the growth in impact resistance of the panel due to the inclusion of EWM. It is equally evident in the figures that the higher impact energies of the EWM panel were noticed with the increment of fiber dosage and cracking and failure stages. For instance, P1 for the FP-1-1 and FP-1-2 panels were 3 and 4 times higher than FP-0-0 panel, while P2 values were about 26 and 42.5 times higher, respectively. This phenomenon may be due to the existence of steel fiber and EWM, along with the crack results in a sufficient improvement in transferring stress along the crack, which in turn improved crack resistance. After the crack formation, the resistance to the panels’ impact depends on fiber and EWM tensile strength and bond strength of the EWM–mortar matrix and fiber mortar matrix. The composite action of EWM and fibers limiting crack opening due to dowel action can hinder the widening of cracks. Therefore, more impacts are needed for debonding between the fibers/EWM and the surrounding mortar matrix. The action of debonding causes fiber/EWM pull-out and the consequent failure of the panel.

#### 4.5.3. Combined Effect of Fibers and Two Layers of EWM on Impact Strength

It is noticeable from Figure 9c and Figure 10c, with an additional layer of EWM, the engrossed impact energies at cracking and failure increases linearly. When the EWM layer increases from 1 to 2, the P1 and P2 increase from 21.5 J and 86.0 J to about 32.4 J and 193.6 J, respectively. Furthermore, fibers at 1% and 2% dosage and two layers of EWM increase the P1 and P2 even higher, which indicates that the fiber addition has a significant influence on the ferrocement panel with a high capacity of impact energies engrossment. For instance,

(i) In comparison with the FP-0-0 panel, P1 and P2 for the FP-2-0 panel were higher by about 3 and 9 times, respectively. By comparing FP-2-0 with FP-1-0 panel, P1 and P2 were higher by about 1.5 and 2.25 times, respectively.
(ii) P1 and P2 for the FP-2-1 panel were 4 and 40.5 times higher as compared to the FP-0-0 panel. Compared with the FP-1-1 panel, P1 and P2 for the same panel were 1.3 and 1.6 times higher, respectively.
(iii) By comparing FP-2-2 with FP-0-0 panel, P1 and P2 were higher by about 5 and 61 times, respectively. Compared with the FP-1-2 panel, P1 and P2 were higher 1.3 and 1.4 times, respectively.

In a nutshell, it may be noticed that the combined action of fiber and two-layer of EWM could improve impact energies at cracking and failure. The capacity of energy engrossment is increased with corresponding increasing dosage of fiber and number of EWM layers. It may be attributed to the fibers, as they are dispersed at random within the mortar matrix; every single fiber serves as a small-scale energy engrossing component during impact. Hence, the two layers EWM panel incorporated between fibers cement can engross better impact energy than two layers EWM panel without fibers counterparts. Before first crack initiation under continuous impacting from the free-falling mass, the fiber and EWM slightly reduced tensile stresses due to their shielding activity, allowing the EWM to absorb some of the applied impact energy, increasing the overall impact capacity of the panel. After crack formation, the EWMs continue their primary job as shock barriers in two different locations and arresting cracks between the three layers. The EWM cut the cracks to the two different thicknesses of the tested panels, which slows down crack propagation and allows for a significant increase in impact resistance. The combined effect of the two actions leads to higher efficiency and better performance of the EWM at the breakdown stage than at the cracking stage.

#### 4.5.4. Combined Effect of Fibers and Three Layers of EWM on Impact Strength

Figure 9c and Figure 10c show the combined effect of fibers and EWMs significant increase in the impact energy engrossment resulting from using three-layer EWM together with fiber.

(i) By looking at the FP-3-0 panel, it is evident that the inclusion of three layers of EWM increased P1 and P2 by 5 and 15.5 times, respectively, over the FP-0-0 panel.
(ii) By comparing FP-3-0 with FP-1-0 and FP-2-0 panel, P1 was higher by about 2.5 and 1.7 times, respectively. Likewise, P2 was 3.9 and 1.7 times higher, respectively.
(iii) P1 for FP-3-1 panel was increased by 6 times as against FP-0-0, 2 times as against FP-1-1, and 1.5 times against FP-2-1 panel. Likewise, P2 was increased by about 64, 2.5, and 1.6 times in respect of FP-0-0, FP-1-1, and FP-2-1, respectively.
(iv) For the FP-3-2 panel, P1 was increased by about 6, 1.5, and 1.2 times compared to FP-0-0, FP-1-2, and FP-2-2 panels, respectively. Likewise, a 92.5, 2.2, and 1.5 times higher P2 were observed, respectively.

The above discussions indicate that the inclusion of three layers of EWM with a 2% dosage of fiber causes a significant enhancement in impact energies. Such implications may be associated with the fiber role in limiting cracks and effective stress transfer through a fiber bridging mechanism. Behavior like this can be ascribed to the same rationale discussed in Section 4.5.3.

#### 4.5.5. Ductility Ratio (DR)

**Figure 11:**
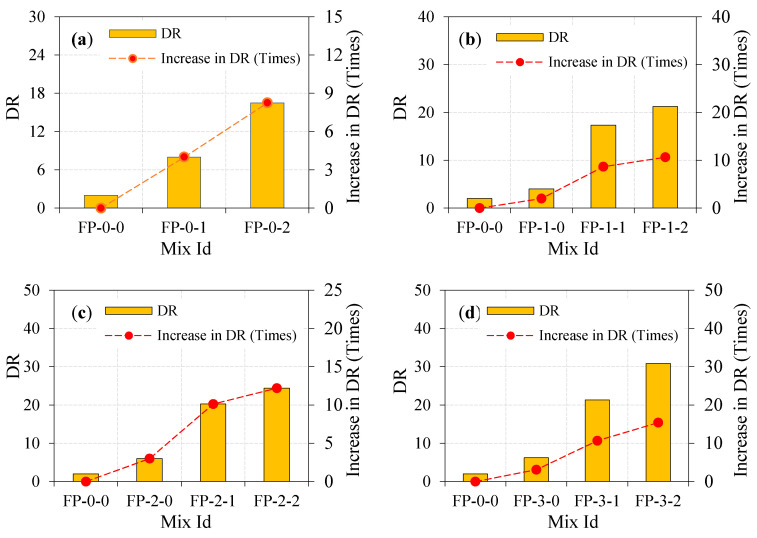
Impact ductility of ferrocement panel under impact load. (**a**) zero layer mesh; (**b**) one layer mesh; (**c**) two layer mesh and (**d**) three layer mesh

The material’s capacity to resist plastic deformation under loading is known as ductility, which is universally applicable with flexural and tensile tests [36]. The ratio of P2 and P1 defines the ductility ratio of the panel. The higher ductility ratio value indicates improved ductility and post cracking behavior of panel under impact load [37,38]. This definition is used by earlier studies [37,38,39] to notice the fiber capability of altering the composite behavior from brittle to ductile against impact loading. It was interesting to note that the significant improvement in DR provided by FP-3-2 was more pronounced than other types of panels. It is noted from Figure 11a, the ductility ratio value ranged between 2 and 16.5 in the case of fibrous panels. This shows the lesser ductility and post crack energy absorption. By looking at Figure 11b, the observed DR values for the one-layer EWM panels ranged between 4 and 21.3, showing more ductility development. Likewise, DR’s improvement was observed in two layers of EWM panels ranging between 6 and 24.4, as shown in Figure 11c. Figure 11d shows that the highest DR was observed in three-layer EWM panels ranging between 6.2 and 30.8. This finding demonstrates that the enormous increase in DR was observed with increased EWM layers and increased fiber dosage. The higher DR is due to the fiber bridging action, which improves the overall impact energy engrossment capacity after cracking. Consequently, the failure is postponed and raises the engrossed number of impacts after cracking.

#### 4.5.6. Failure Mode of Ferrocement Panel

**Figure 12:**
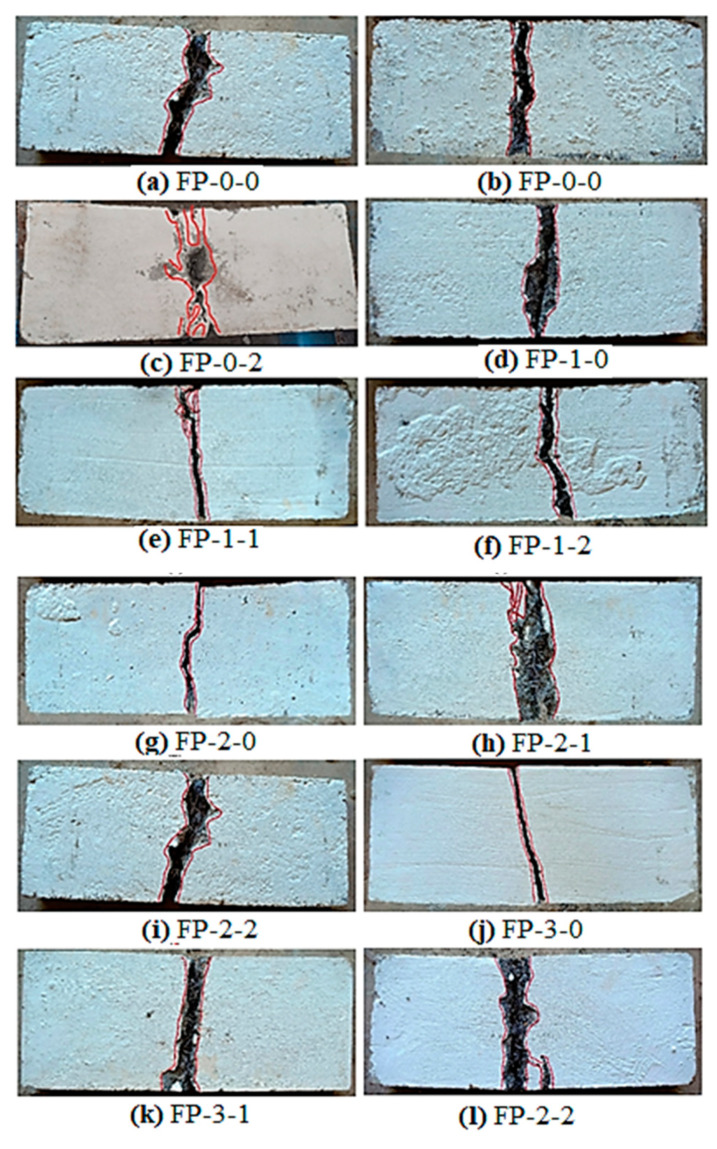
Failure mode of all teste ferrocement panel under impact load.

The noticed failure modes of all tested ferrocement panels are shown in Figure 12a–l. The FP-0-0 panel showed lesser resistance to impact compared to fibrous panels. It may be noticed that the failure of these panels happened all of a sudden after a few impacts from the initial crack formation, showing the brittle failure but broken into two parts. As the crack resistance capacity was reached, cracks formed at the panel’s bottom surface, and it became more expansive and propagated to its top surface under repeated impact. This is the most widely known failure mode in the ferrocement panels, which agrees with many earlier studies available [4,40]. This brittle failure is due to a lack of bridging element that retains the bonding along each developed crack. On the other hand, the FP-1-0 and FP-2-0 panels also exhibited sudden failure, absorbing only more impacts than the FP-0-0 panel.

**Figure 13:**
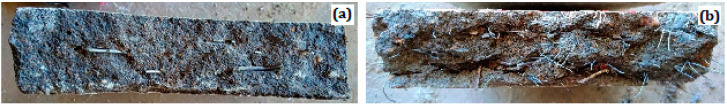
Appearance of failure plane. (**a**) two layer mesh (**b**) three layer mesh.

All fibrous panels showed different behavior where the impact of energy engrossment was improved significantly due to EWM and fiber inclusion. As a result of being an engrossing higher number of impacts, a crack was formed at the panel’s bottom surface. Cracks of these opened more expansive as the increased number of impacts extended to the top surface. The fiber bridging action continues on both sides of cracks and, together with EWM, restrict the crack expansion towards the top surface. Later, the debonding occurs due to the gradual loss of bonding between the fiber/EWM and surrounding mortar matrix resulting in panel failure by fiber pull out and EWM breakage is shown in Figure 13. This type of failure is called a ‘ductile failure’ because it absorbs more impact energies and delays failure after cracking. Such occurrences can be assumed in consequence of two mechanisms: orientation of fiber and bonding between the ESW/fiber and surrounding mortar matrix.

**Figure 14:**
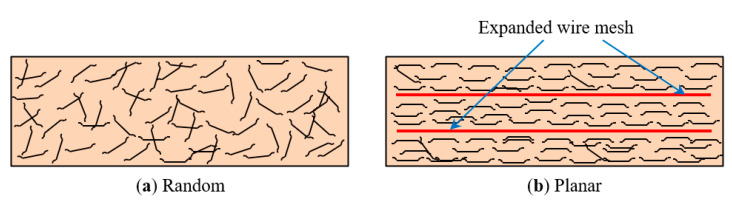
Distribution of fibers in ferrocement panel: (**a**) random and (**b**) planar.

The FP-0-0 panel has a 3D orientation, while the fiber alignment for other panels was planar is shown in Figure 14. The fibers with 3D orientation had a trend of declining performance against impact load, while fibers with planar alignment increase it. This behavior is consistent with earlier studies [41]. Moreover, bonding behavior between the ESW/fiber and surrounding mortar matrix contributes significantly to higher impact energy engrossment. As a result of being a planar orientation of fiber, fibrous panels could engross more energy than non-fibrous ones.

#### 4.5.7. Failure Mechanism of Panel under Impact Load

**Figure 15:**
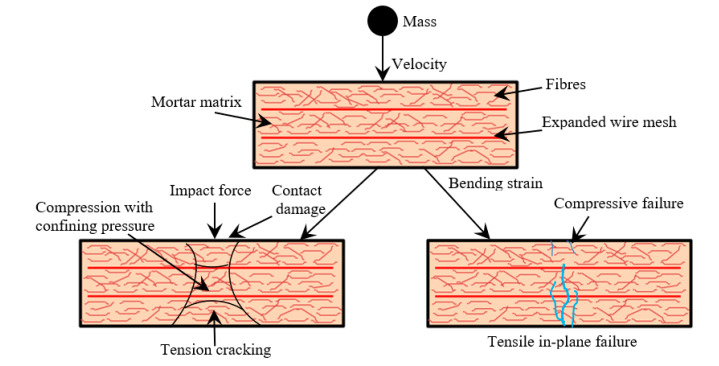
Schematic description of activated failure mechanism under the repeated falling mass impact.

Figure 15 depicts the schematic description of the fracture mechanism of the ferrocement panel under the repeated falling mass impact. The fracture mechanism sequence is described in four phases: damage in point of contact, failure of mortar matrix, failure of fiber/EWM, and debonding. The mortar matrix will be broken apart along the impact force direction, such as contact damage. Internal debonding of the panel as a result of transverse shear strain/stress [42]. Fiber-matrix and EWM-matrix failure resulting from compression bending. Fiber and EWM are debonding to the surrounding matrix due to tensile bending at the bottom face. Debonding of fibers and EWM is a severe incident in the process of fracture, which largely influenced the panel’s strength and integrity. The aforesaid fracture effects’ timeframe is minimal, and therefore it is tough to spot at service. After cracking, a significant amount of initial kinetic energy transfer occurred from the mortar matrix to fiber and EWM. This indicates that fibers and EWM can arrest cracks and dissipate more energy to the surrounding mortar matrix. Once fibers and EWM can no longer limit crack development, debonding happens. The panel will fail after the distribution of stress in the ferrocement panel during impact.

## 5. Conclusions

An extensive laboratory investigation was conducted on the flexural and impact response of ferrocement panel. Different panels were formulated with varying amounts of steel fiber and expanded wire mesh (EWM) layers. The analysis and interpretation of experimental outcomes obtained from the current study have let the following conclusions be reached:

1. The highest compressive strength at 28 days was 70.8% and 50.5% for the mortar cube incorporating 2% and 1% dosage of fiber, respectively, compared to non-fibrous mortar. This phenomenon was due to fiber addition with high contents, which triggered micro-cracks formation before the ultimate crack and enhanced resistance to crack development and propagation.
2. The increasing number of EWM layers and steel fibers significantly improved the ultimate load capacity, flexural strength, and ductility index of the ferrocement panels. However, the best contribution comes from the panel, comprising three layers of EWM with 2% steel fibers.
3. The ultimate load of the ferrocement panels increased significantly as the number of EWM layers and fiber dosage increased. Regarding the ultimate load and mid-span deflection, the best contribution comes from the FP-3-2, followed by FP-2-2 panels. These panels exhibited 149% and 126% higher ultimate load than FP-0-0 panels with the corresponding deflection of 4.4 mm and 4.66 mm, respectively.
4. The mortar matrix influenced the width of the crack. Adding 2% steel fibers in a mortar and three-layers EWM of the fabricated ferrocement panel (FP-3-2) enhanced resistance to crack and flexural capacity. Crack opening width is reduced by about 68% compared to that of the reference panel (FP-0-0).
5. The number of EWM inclusion and steel fibers resulted in an additional impact resistance improvement at cracking and failure stages for all types of ferrocement panels. Comparing FP-3-2 with FP-0-0 panel, the use of three-layer of EWM and 2% fiber resulted in higher impact records by approximately 6 times at cracking (P1) and 92.5 times at failure (P2). This additional input of impact resistance is attributed to EWM and fibers’ combined action as restriction barriers against crack propagation across the subsequent ferrocement layers.
6. Increase in impact resistance and ductility ratio is attributed mostly to the high content of steel fibers and EWMs in the ferrocement panels, which changed the response from brittle to ductile. Fibers arrest cracks during their initiation under impact loads resulting in higher engrossed energy at this level. In contrast, fiber bridging action’s ultimate efficiency was reached after crack initiation, where fibers carry the tensile stresses across the cracks preventing their propagation and widening. Therefore, the obtained impact enhancements at failure were noticeably higher than those obtained at cracking.
7. With such superior impact resistance and ductile response, this ferrocement panel can be a positive choice for partitioning structures, footbridges, roof shells, silos, swimming pools, and manhole covers subjected to repeated impacts.

Normally, mesh inserted ferrocement and fiber incorporation fulfils the demands of impact resistance. It is suggested that ferrocement panel reinforced with other types of fibers (crimped, polypropylene, glass, basalt, etc.) and other types of wire meshes (square woven, square welded, hexagonal, etc.) to be explored further under various states of the environment and fire conditions prior to being used safely in field applications. The research objective was to get an idea of the practicability of the proposed ferrocement from the prospective viability of impact behavior. The results in this paper are based on apparatus used in the laboratory in a tropical climate (India). While this experimental setup and technique were used by earlier researchers to examine the impact behavior of concrete, this may take reliability into consideration, but they cannot precisely signify the same conditions in the field.

## Figures and Tables

**Table 1:** Details of mixing composition used in the fabrication of ferrocement panel.

Mix	Cement (kg/m^3^)	Sand (kg/m^3^)	Water (kg/m^3^)	Fiber Dosage (wt. %)	WRA (%)	Number of Mesh	Thickness of Layer (mm)			
							1	2	3	4
FP-0-0	700	1400	280	0	0.7	0	50	-	-	-
FP-0-1	700	1400	280	1	1	0	50	-	-	-
FP-0-2	700	1400	280	2	1.3	0	50	-	-	-
FP-1-0	700	1400	280	0	0.7	1	25	25	-	-
FP-1-1	700	1400	280	1	1	1	25	25	-	-
FP-1-2	700	1400	280	2	1.3	1	25	25	-	-
FP-2-0	700	1400	280	0	0.7	2	15	20	15	-
FP-2-1	700	1400	280	1	1	2	15	20	15	-
FP-2-2	700	1400	280	2	1.3	2	15	20	15	-
FP-3-0	700	1400	280	0	0.7	3	10	15	15	10
FP-3-1	700	1400	280	1	1	3	10	15	15	10
FP-3-2	700	1400	280	2	1.3	3	10	15	15	10

WRA: Water reducing agent.

**Table 2:** Load and deflection comparison of ferrocement panel for flexural test.

Mix Id	Load (kN)		Deflection (mm)		Ductility Index δ_u_/δi
	W_i_	W_u_	δ_i_	δ_u_	
FP-0-0	1.15	3.25	1.95	3.83	2.0
FP-0-1	1.40	5.50	1.86	4.26	2.3
FP-0-2	1.45	6.20	1.85	5.12	2.8
FP-1-0	1.56	5.35	2.56	5.76	2.3
FP-1-1	1.59	6.20	1.90	4.85	2.6
FP-1-2	1.62	7.15	1.42	4.02	2.8
FP-2-0	1.60	5.65	2.46	6.22	2.5
FP-2-1	1.68	6.30	1.85	5.06	2.7
FP-2-2	1.78	7.35	1.62	4.66	2.9
FP-3-0	1.71	6.55	2.63	6.89	2.6
FP-3-1	1.77	7.2	1.96	5.51	2.8
FP-3-2	1.83	8.1	1.74	4.96	2.9

**Table 3:** Experimental finding from a drop weight test.

Mix Id	Number of Impacts		Impact Energy (J)		Impact Ductility Index (IDI)
	Z1	Z2	P1	P2	
FP-0-0	1	2	10.8	21.5	2.0
FP-0-1	2	16	21.5	172.1	8.0
FP-0-2	2	33	21.5	354.9	16.5
FP-1-0	2	8	21.5	86.0	4.0
FP-1-1	3	52	32.3	559.2	17.3
FP-1-2	4	85	43.0	914.1	21.3
FP-2-0	3	18	32.3	193.6	6.0
FP-2-1	4	81	43.0	871.0	20.3
FP-2-2	5	122	53.8	1311.9	24.4
FP-3-0	5	31	53.8	333.4	6.2
FP-3-1	6	128	64.5	1376.5	21.3
FP-3-2	6	185	64.5	1989.4	30.8

## Nomenclature

EWM	Expanded wire mesh
w/c	Water–cement ratio
ASTM	American Society for Testing and Materials
ACI	American Concrete Institute
WRA	Water reducing agent
FP	Ferrocement panel
LVDT	Linear variable displacement transducers
σ	Flexural strength, MPa
L	Support span, mm
b	Tested panel width, mm
d	Tested panel depth, mm
W	Applied load, N
N	Recorded impacts number
m	Steel-ball weight
g	Gravitational acceleration
H	Vertical falling distance
Z1	Number of impacts leading to cracking failure
Z2	Number of impacts leading to failure
P1	Absorbed energies at cracking
P2	Absorbed energies at failure
W_i_	Initial crack load
W_u_	Ultimate load
δ_i_	Initial crack deflection
δ_u_	Ultimate load deflection
IDI	Impact ductility index
DR	Ductility ratio
